# Effects of wearable lower-limb robotic exoskeleton–assisted gait training on balance, walking, and activities of daily living after stroke: a systematic review and meta-analysis

**DOI:** 10.3389/fneur.2026.1818981

**Published:** 2026-10-16

**Authors:** Zezhong Zhang, Xinyuan Zhang, Sujie Mao, Lei Cao, Zilong Wang, Jingtao Wu, Wanli Zang

**Affiliations:** 1 College of Physical Education, Harbin University, Harbin, China; 2 School of Physical Education, Soochow University, Suzhou, Jiangsu, China; 3 Postgraduate School, Harbin Sport University, Harbin, Heilongjiang, China; 4 School of Physical Education, Jimei University, Xiamen, Fujian, China; 5 School of Physical Education, Leshan Normal University, Leshan, Sichuan, China

**Keywords:** balance, exoskeleton-assisted gait training, Fugl–Meyer, meta-analysis, stroke, systematic review, walking independence, wearable robotic exoskeleton

## Abstract

**Background:**

Wearable lower-limb robotic exoskeleton–assisted gait training encompasses heterogeneous overground and treadmill/platform-based implementations. Comparative effects remain uncertain. We synthesised evidence from randomised controlled trials (RCTs) on walking, balance, lower-limb motor function, and activities of daily living (ADL).

**Methods:**

We searched PubMed, Embase, the Cochrane Central Register of Controlled Trials, Web of Science Core Collection, and China National Knowledge Infrastructure from inception to February 26, 2026. We included RCTs comparing wearable lower-limb robotic exoskeleton–assisted gait training (overground and/or treadmill/platform-based) with usual rehabilitation or other controls. Effects were pooled using random-effects models with restricted maximum likelihood estimation and Hartung–Knapp adjustment; 95% prediction intervals were reported when applicable. Certainty was assessed using the Grading of Recommendations Assessment, Development and Evaluation approach, and publication bias was evaluated for outcomes with ≥10 studies.

**Results:**

Fourteen RCTs (*n* = 565) were included. Pooled between-group estimates favoured intervention groups for walking independence measured by the Functional Ambulation Category (FAC; mean difference [MD] = 0.39, 95% confidence interval [CI] 0.15–0.63), balance measured by the Berg Balance Scale (BBS; MD = 1.66, 95% CI 0.52–2.81), and lower-limb motor function measured by the Fugl–Meyer Assessment of the Lower Extremity (FMA-LE; MD = 2.40, 95% CI 1.63–3.16). The 6-Minute Walk Test estimate favoured intervention groups (MD = 19.83, 95% CI 2.21–37.45), although its prediction interval crossed the null. No between-group differences emerged for walking speed, mobility, or ADL. Certainty was moderate for FAC, BBS, and FMA-LE and low to very low for other outcomes. No small-study effects were detected for BBS (k = 11; *p* > 0.05).

**Conclusion:**

Across heterogeneous exoskeleton-assisted gait-training interventions, pooled estimates favoured intervention groups for walking independence, balance, and lower-limb motor function. However, most prediction intervals crossed the null, certainty for other outcomes was low to very low, and inconsistent reporting limited analyses of training environment and precluded granular analyses of body-weight support, assistance strategy, patient engagement, comparator content, dose, and patient selection. These estimates reflect heterogeneous intervention–comparator packages and cannot determine whether differences are attributable to the robotic device category itself or support specific prescribing recommendations. Larger, stratified RCTs with standardised reporting and longer follow-up are needed to identify influential intervention and patient characteristics.

**Systematic review registration:**

https://www.crd.york.ac.uk/PROSPERO/view/CRD420261327255

## 1 Introduction

Stroke remains one of the leading causes of death and long-term disability worldwide, and its overall burden has remained high over the past decades. Post-stroke declines in walking ability, balance control, and activities of daily living (ADL) are key factors limiting social participation and quality of life, and constitute important and quantifiable clinical targets in neurorehabilitation (1–3). In rehabilitation practice, early restoration of safe, effective, and sustainable walking function is considered an important pathway to promote independent living and reduce caregiving burden (3).

However, conventional gait training often faces practical constraints in the delivery of sufficient, safe, and repeatable gait practice. In individuals in the early post-stroke stage or those with moderate-to-severe hemiparesis, achieving high-repetition walking practice with sufficient step counts can be challenging. Therapists also experience substantial physical demands during manually assisted gait training, which may limit the consistency of training intensity over time. In addition, fall risk and compensatory gait patterns may compromise training quality. Accordingly, electromechanical/robot-assisted gait training (RAGT) has been developed to facilitate repetitive gait practice and provide controlled assistance. Among these approaches, wearable lower-limb robotic exoskeleton systems represent a distinct category in which robotic assistance is delivered through wearable devices during gait practice. Systematic evidence suggests potential benefits for walking-related outcomes after stroke; however, treatment effects vary substantially according to device characteristics, training strategies, patient characteristics, and rehabilitation contexts (4, 5). Furthermore, a dose–response relationship may exist between rehabilitation dose/intensity and functional recovery, highlighting the potential importance of sufficient training intensity, repetition, and task specificity in neurorehabilitation (6).

In recent years, wearable lower-limb robotic exoskeletons—implemented in overground and/or treadmill-based gait training contexts—have attracted increasing attention as a potential approach for post-stroke gait rehabilitation. Compared with conventional rehabilitation approaches, these systems may provide additional robotic assistance and structured gait practice, potentially facilitating repetitive and task-specific training. However, the clinical implications of these potential advantages may vary according to device characteristics, training environment, assistance strategies, and patient interaction with the system. Systematic reviews and meta-analyses focusing on wearable robotic exoskeleton-assisted gait training suggest potential improvements in walking ability, walking speed, balance, endurance, and some functional outcomes; however, the magnitude and consistency of effects remain variable across outcome domains, intervention characteristics, and study designs (7–10). Meanwhile, randomised controlled trials (RCTs) and pragmatic studies have accumulated rapidly in recent years, providing additional evidence regarding implementation feasibility and clinical efficacy of exoskeleton-assisted rehabilitation (11–13).

From an evidence synthesis perspective, previous reviews have evaluated broader robotic-assisted gait training categories, including wearable exoskeletons, treadmill-based robotic systems, and other robotic rehabilitation devices, encompassing different robotic technologies, training environments, delivery approaches, comparators, and outcome selections (4, 7–10). Although these reviews provide important evidence regarding robotic-assisted rehabilitation after stroke, the evidence specifically focusing on wearable lower-limb robotic exoskeleton-assisted gait training remains incompletely characterised because previous evidence syntheses have often combined heterogeneous robotic interventions with different device architectures, training environments, and assistance strategies. Furthermore, wearable exoskeleton-assisted training itself includes diverse clinical applications, such as overground and treadmill/platform-based training, with variations in assistance strategies, external support requirements, and rehabilitation contexts. These differences may contribute to variability in reported effects across functional outcomes and rehabilitation contexts. Therefore, an updated systematic review focusing specifically on wearable lower-limb robotic exoskeleton-assisted gait training is needed to evaluate whether treatment effects differ across clinically relevant rehabilitation domains and to determine the certainty and variability of the available evidence (9, 10).

Therefore, we conducted a systematic review and meta-analysis focusing on wearable lower-limb robotic exoskeleton–assisted gait training (overground and/or treadmill-based) in people with stroke to evaluate its effects across clinically relevant rehabilitation domains, including walking independence, walking performance, balance and mobility, lower-limb motor function, and ADL (14–16). Given that wearable exoskeleton-assisted training encompasses diverse technological and clinical implementations, we aimed to evaluate outcome-specific effects across functional domains while interpreting findings in the context of variations in intervention characteristics and rehabilitation contexts. In addition, considering the clinical and technical heterogeneity of available RCTs and their limited sample sizes, we assessed the certainty and variability of the current evidence using approaches that account for between-study uncertainty (17–19).

## 2 Methods

This systematic review and meta-analysis was designed, conducted, and reported in accordance with the Preferred Reporting Items for Systematic Reviews and Meta-Analyses (PRISMA 2020) guidelines. The protocol was prospectively registered in PROSPERO (registration number: CRD420261327255).

### 2.1 Information sources

We systematically searched PubMed, Embase, the Cochrane Central Register of Controlled Trials via the Cochrane Library, China National Knowledge Infrastructure, and Web of Science Core Collection from inception to February 26, 2026. To identify ongoing or unpublished trials, we also searched ClinicalTrials.gov on the same date, using the topic of RCT in people with stroke involving wearable robotic exoskeleton–assisted gait/walking training for the lower limb.

All databases were searched independently via their native search interfaces; no updated search was performed after the final search date, and no email alerts were set. During full-text assessment, we performed backward reference checking of the included studies and relevant systematic reviews to supplement the search; screening was completed on February 26, 2026. We did not conduct forward citation tracking, nor did we additionally search grey literature databases, preprint platforms, or Google Scholar. When necessary, we contacted corresponding authors to obtain missing information or outcome data.

### 2.2 Search strategy

The search strategy combined controlled vocabulary (e.g., Medical Subject Headings and Emtree) and free-text keywords, and was constructed around terms related to stroke, lower-limb exoskeleton/wearable robotic exoskeleton, exoskeleton-assisted gait training, and RCTs. We did not use published search filters (e.g., validated RCT filters); study design was restricted using a combination of controlled vocabulary terms and free-text terms. The search strategy was newly developed and was not directly adapted from, or reused from, previous systematic reviews. Searches were limited to studies published in English or Chinese and conducted in humans [adults (≥18 years)]. Development and reporting of the search strategy followed the Preferred Reporting Items for Systematic Reviews and Meta-Analyses extension for literature searches. The full search strings for each database and information source (including all search terms, Boolean operators, limits, and the final search date) are provided in Appendix A. The search strategy was not formally peer reviewed.

### 2.3 Eligibility criteria

Studies meeting all of the following criteria were included in this systematic review and meta-analysis:

1. *Study design*: RCTs (including parallel designs or crossover designs from which first-period data could be extracted).
2. *Participants*: Adults aged ≥18 years with a clinical diagnosis of stroke (ischaemic or haemorrhagic) and post-stroke lower-limb motor impairment and/or limited walking ability. No restrictions were placed on sex. Lower-limb impairment/walking limitation could be defined based on the original study’s eligibility description, such as hemiparesis/lower-limb muscle weakness, inability to walk independently or Functional Ambulation Category (FAC) below the independent walking level, impaired 10-Meter Walk Test (10MWT)/walking speed, or a need for assistive devices or assistance from another person.
3. *Interventions*: The experimental group received gait training involving a wearable lower-limb robotic exoskeleton or robotic orthosis (eg, powered exoskeleton, soft exosuit, unilateral/bilateral lower-limb exoskeleton) as the primary technological component of the intervention. In this review, wearable lower-limb robotic exoskeleton-assisted gait training was defined as gait training using a robotic device physically coupled to the participant’s lower limbs. Given the diversity of available robotic systems, wearable lower-limb robotic exoskeleton-assisted gait training was considered an intervention category rather than a single device-specific therapy. Eligible interventions included exoskeleton-assisted walking training delivered in different training contexts, including overground walking and treadmill/training-platform-based gait training, either alone or as an adjunct to usual rehabilitation. Both overground and treadmill/platform-based training were eligible when the robotic device provided direct lower-limb assistance during gait practice. To characterize intervention heterogeneity, relevant intervention features were extracted when reported, including training environment (overground versus treadmill/platform-based), use of body-weight support or external assistance (eg, harness, handrails, walkers, or crutches), robotic configuration (eg, unilateral versus bilateral assistance), assistance/control strategy, training dose, and therapist involvement. Co-interventions with usual rehabilitation (eg, physiotherapy, gait training, or balance training) were allowed, provided that the robotic exoskeleton component represented the primary differentiating component between groups.
4. *Comparators*: The control group received rehabilitation interventions without an active exoskeleton component, including usual rehabilitation/usual physiotherapy, conventional gait training, usual walking training (including walkers/manual assistance), dose-matched rehabilitation training, or sham/placebo device conditions. Comparator interventions were considered according to the reported rehabilitation context, including training content, intensity/dose, and therapist involvement when available.
5. *Outcomes*: Studies had to report at least one prespecified outcome with extractable data (at minimum, baseline and at least one post-intervention/follow-up time point). Outcomes included, but were not limited to, FAC, 10MWT, 6-Minute Walk Test (6MWT), Berg Balance Scale (BBS) or other equivalent balance scales, Timed Up and Go test (TUG), Rivermead Mobility Index (RMI), Fugl–Meyer Assessment of the Lower Extremity (FMA-LE), Motricity Index–Lower Extremity (MI-LE), Barthel Index (BI), Modified Barthel Index (MBI), Korean Modified Barthel Index (K-MBI), and gait symmetry–related indicators (eg, step length/step time symmetry or symmetry index [SI]).
6. *Other restrictions*: only human studies published in English or Chinese were included.

Studies meeting any of the following criteria were excluded:

1. Non-randomised designs (eg, non-randomised controlled studies, pre–post studies without a control group, cohort/case–control studies, case series, reviews/commentaries).
2. Participants were not people with stroke, or the population comprised mixed neurological conditions and stroke-specific data could not be extracted separately; or participants were children/adolescents (<18 years).
3. Ineligible intervention: Ineligible interventions included those that did not involve a wearable lower-limb robotic exoskeleton or robotic orthosis, or in which the robotic component was not the primary differentiating element between intervention and control groups. Treadmill-based interventions were eligible only when the participant wore a lower-limb robotic exoskeleton/robotic orthosis during gait training. Non-wearable electromechanical gait systems (eg, end-effector devices or platform-based robotic systems without a wearable lower-limb robotic component) were excluded.
4. Inappropriate comparator: No comparable control group, or the control group received the same exoskeleton-assisted intervention, preventing estimation of the incremental effect of exoskeleton-assisted training.
5. No outcomes of interest were reported, or extractable statistics were unavailable (eg, mean and standard deviation, change values and their variance, convertible median and interquartile range), and could not be obtained from the full text, Supplementary materials, or by contacting authors.
6. Full text was unavailable; or duplicate publications/multiple reports from the same trial were present, in which case only the most complete or most recent report was retained.

### 2.4 Study selection process

Two reviewers independently screened all records retrieved from the searches. In the first stage, relevance and study design were assessed based on titles and abstracts; in the second stage, full texts were evaluated and final inclusion was determined according to prespecified eligibility criteria. Discrepancies were resolved through discussion; if consensus could not be reached, a third reviewer adjudicated. The study selection process was documented and presented using a PRISMA 2020 flow diagram. No automation tools were used in study selection.

### 2.5 Data collection process

Two reviewers independently extracted data using a standardised data extraction form that had been piloted in advance. Disagreements during data extraction were resolved through discussion, with adjudication by a third reviewer when necessary. For each prespecified outcome, we extracted data at baseline and at the end of the intervention (extracting post-intervention values and/or change scores as reported). In the primary pooled analyses, we prioritised the end-of-intervention assessments.

In parallel, we extracted risk-of-bias–related information for each included study to support subsequent risk-of-bias assessment and Grading of Recommendations Assessment, Development and Evaluation (GRADE) judgements of evidence certainty. When key data were missing or unclear, we attempted to contact study authors; if additional information could not be obtained, analyses were conducted using the data as reported in the articles. No automation tools were used for data extraction.

### 2.6 Data items (outcomes)

We extracted outcome data according to prespecified outcome domains and measurement instruments, and verified the scoring direction and units for each scale/indicator to ensure consistent interpretation; when necessary, outcome direction was harmonised (eg, for measures where shorter time indicates better performance, the direction was transformed during interpretation and pooling to align with other outcomes). Outcomes of interest included:

1. Walking and gait performance: FAC; 10MWT (reported in seconds or m/s); 6MWT (reported in metres); and gait symmetry–related indicators (eg, step length symmetry, step time symmetry, symmetry index/ratio, as defined in the original studies).
2. Balance and mobility: BBS; TUG; RMI.
3. Lower-limb motor function/impairment level: FMA-LE (including short-form versions, extracted as reported in the original studies); MI-LE.
4. ADL: BI; MBI and its regional/language versions (eg, K-MBI), extracted according to the version used in the original studies.

For each outcome, we extracted assessment time points and data at baseline and post-intervention (end of intervention; extracting post-intervention values and/or change scores as reported), and recorded group sample sizes and means with standard deviations and standard errors.

### 2.7 Data items (other variables)

In addition to outcome data, we extracted the following information on other variables:

1. Study characteristics

First author, publication year, country/setting (eg, inpatient rehabilitation, outpatient, community, laboratory), study design (restricted to RCTs; parallel/crossover), recruitment source, information on randomisation and allocation concealment/blinding (if reported), sample size in each group (randomised and completed/analysed), follow-up duration, funding source, and conflicts of interest statements when reported.

2. Participant baseline characteristics

Age, sex distribution, stroke type (ischaemic/haemorrhagic) and lesion side, time since stroke/stage (acute/subacute/chronic, recorded as defined in the original studies), baseline functional status (eg, baseline FAC, 10MWT/walking speed, use of assistive devices or need for assistance from another person), severity of lower-limb motor impairment (eg, baseline FMA-LE/MI-LE), key exclusion criteria such as comorbidities and cognitive/communication impairments, and baseline comparability between groups.

3. Intervention and comparator characteristics

Because wearable lower-limb robotic exoskeleton-assisted gait training represents a heterogeneous intervention category rather than a single therapeutic modality, intervention characteristics were extracted according to a structured taxonomy. The extracted intervention features, including device characteristics, training environment, robotic assistance strategy, external support requirements, training dose, and comparator characteristics, are summarized in Supplementary Table S1.

a. Device characteristics

Exoskeleton type (rigid versus soft), unilateral versus bilateral assistance, device model/brand (when available), robotic architecture, and lower-limb joints assisted.

b. Training environment and delivery context

Training environment (overground versus treadmill/platform-based training), rehabilitation setting (inpatient, outpatient, community, or laboratory), and whether training was conducted with additional external support (eg, body-weight support harness, handrails, walkers, crutches, or therapist assistance).

c. Robotic assistance and control strategy

Assistance mode/control strategy (eg, active, passive, adaptive, or triggered assistance), assistance level or relevant parameters (when reported), and indicators of patient engagement or active contribution during gait practice.

d. Training dose and intervention content

Intervention dose, including training frequency, session duration, total intervention duration, total training volume, and step counts when available. Additional intervention characteristics were extracted when reported, including progression rules, walking practice components (eg, turning, balance challenges, or variable walking tasks), strength or balance components, co-interventions with usual rehabilitation, and whether intervention dose was matched between groups.

e. Comparator characteristics

Comparator interventions were extracted separately to characterize the rehabilitation context against which exoskeleton-assisted training was compared. Extracted variables included type of control intervention, rehabilitation content (eg, conventional physiotherapy, gait training, or balance training), training dose and intensity when reported, therapist involvement, and whether the comparator was dose-matched.

4. Training intensity, adherence, and safety outcomes

Intensity/load measures (including but not limited to rating of perceived exertion [RPE], heart rate, step counts/cadence, assistance proportion, torque or power parameters, and speed settings, when reported), adherence/completion rate, reasons for dropout and loss to follow-up, and adverse events (eg, falls, skin pressure injury/pain, fatigue, or joint discomfort) were extracted.

### 2.8 Risk of bias assessment

We used the Cochrane Risk of Bias tool (RoB 1) and, based on the Cochrane Handbook (version 5.1.0), assessed risk of bias at the study level for included RCTs. The assessed domains included random sequence generation, allocation concealment, blinding of participants and personnel, blinding of outcome assessment, incomplete outcome data, selective reporting, and other sources of bias. Each domain was judged as low risk, high risk, or unclear risk. Overall risk of bias was determined as follows: overall low risk if all domains were low risk; overall high risk if one or more domains were high risk; otherwise, overall unclear risk. Two reviewers performed the assessments independently; discrepancies were resolved through discussion, with adjudication by a third reviewer when necessary. When additional information was required, we contacted study authors to clarify issues relevant to risk-of-bias judgements. No automation tools were used for risk-of-bias assessment.

### 2.9 Effect measures

Prespecified outcomes in this review were primarily continuous. For outcomes reported using the same measurement tool and on the same scale/metric across studies, we pooled effects using the mean difference (MD) with 95% confidence intervals (CI), for example: 10MWT (m/s), 6MWT (m), BBS (points), TUG (s), RMI (points), FMA-LE (points), MI-LE (points), BI (points), and MBI/K-MBI (points). When different scales were used to assess the same construct and pooling was clinically justifiable, we used the standardised mean difference (SMD; Hedges’ g) with 95% CI. Gait symmetry indicators (eg, step length/step time symmetry or SI) may vary in definition and calculation across studies. If definitions were consistent or could be reasonably converted to a common metric, we pooled using MD; if metrics differed but reflected the same symmetry construct and were comparable, we pooled using SMD; otherwise, results were summarised narratively. For FAC, as all included studies reported data as mean ± standard deviation, we treated it as a continuous variable and pooled using MD with 95% CI. For each outcome, we prioritised post-intervention (end-of-intervention) assessments as the primary time point for pooling. Data extraction prioritised post-intervention values; if only change-from-baseline values were reported, we used them as reported. If change scores were not directly reported but could be derived from pre- and post-intervention summary statistics, we calculated them using standard formulas; otherwise, post-intervention values were used. All meta-analyses reported 95% CI; when the number of included studies was ≥3, we additionally reported the 95% prediction interval. We did not prespecify unified thresholds for “effect size” categories; interpretation was primarily based on the clinical meaning of the original scales (for MD) and evidence certainty (GRADE).

### 2.10 Synthesis methods

#### 2.10.1 Eligibility for quantitative synthesis by outcome

For each prespecified outcome, studies were eligible for quantitative synthesis if they were RCTs, included at least one comparison of “wearable lower-limb robotic exoskeleton intervention vs control”, and reported extractable data for the outcome at baseline and post-intervention (end of intervention; post-intervention values and/or change-from-baseline values). If fewer than two studies were available for a given outcome, no meta-analysis was performed and findings were summarised narratively.

#### 2.10.2 Data preparation before pooling

Before pooling, we verified the definition, measurement units, and scoring direction of each outcome to ensure consistent interpretation of effect estimates. When required summary statistics were not directly reported, we attempted to derive them from available information (eg, standard errors or 95% confidence intervals) using standard formulas; if derivation was not possible, we contacted original study authors for the data. If key statistics remained unavailable, the outcome was not included in the pooled analysis. When the same outcome was reported using different units/formats (eg, 10MWT reported in seconds or m/s), and no harmonisation was performed, results were pooled separately by unit or summarised narratively.

#### 2.10.3 Tabular summary and visual presentation

Study characteristics and extracted outcome data were summarised in tables. Meta-analysis results were presented using forest plots. Risk-of-bias judgements were summarised both graphically (eg, traffic-light plots) and in tables.

#### 2.10.4 Methods for synthesis

Studies were grouped by outcome domain and measurement tool and pooled separately. We conducted independent meta-analyses for the following outcome domains:

- Walking ability and gait performance: FAC, 10MWT (analysed separately as “s” or “m/s”), and 6MWT;
- Balance and functional mobility: BBS, TUG, and RMI;
- Lower-limb motor function/impairment level: FMA-LE and MI-LE;
- ADL: BI, MBI and its versions (eg, K-MBI), pooled by scale when necessary or using SMD;
- Gait symmetry (when applicable): step length/step time symmetry and symmetry index/ratio (eg, SI). If definitions were consistent or could be reasonably converted to a common metric, quantitative synthesis was undertaken; otherwise, findings were summarised narratively.

When multiple time points were reported, the primary pooled analysis prioritised post-intervention assessments. Meta-analyses were conducted in R (meta package). For continuous outcomes, MD was used when the same scale/unit was applied across studies; SMD (Hedges’ g) was used when different tools assessed the same construct with clinical comparability. All effect estimates were reported with 95% CI. Given anticipated clinical and methodological differences, all meta-analyses used a random-effects model (20). Between-study variance (τ^2^) was estimated using restricted maximum likelihood (REML) (21). More robust confidence intervals were calculated using the Hartung–Knapp–Sidik–Jonkman method (17). Statistical heterogeneity was quantified using I^2^ and τ/τ^2^ statistics. When at least three studies were available for an outcome, we calculated 95% prediction intervals for the random-effects meta-analysis using the approach described by Nagashima and colleagues and implemented in the meta framework (via the pimeta dependency) (19, 22).

#### 2.10.5 Exploration of heterogeneity

Because most pooled analyses included a limited number of studies, formal investigations of sources of heterogeneity were considered potentially underpowered. Therefore, meta-regression analyses and formal tests of interaction were not performed. When sufficient studies were available, exploratory subgroup analyses were conducted to examine potential clinical sources of heterogeneity. Specifically, training environment (overground versus treadmill/platform-based training) was evaluated as a subgroup factor for outcomes with adequate data availability. Subgroup analyses were considered exploratory due to the limited number of available studies.

#### 2.10.6 Sensitivity analyses

Sensitivity analyses were conducted for outcomes where pooled estimates were considered potentially influenced by individual studies or substantial heterogeneity. Leave-one-out analyses were performed by sequentially removing each individual study and recalculating the pooled effect estimates to evaluate the influence of individual studies on the overall findings.

### 2.11 Assessment of reporting bias

When at least 10 studies were available for a specific outcome, potential small-study effects and publication bias were assessed using contour-enhanced funnel plots and Egger’s regression test. Outcomes with fewer than 10 studies were not evaluated because funnel plots and regression tests have limited reliability in small samples.

### 2.12 Certainty of evidence assessment

We used the GRADE approach to assess the certainty of evidence for each prespecified outcome, considering five domains: risk of bias, inconsistency, imprecision, indirectness, and publication bias when applicable (23). Certainty was classified as high, moderate, low, or very low. Two reviewers independently conducted the GRADE assessments; disagreements were resolved through discussion, with adjudication by a third reviewer when necessary.

## 3 Results

### 3.1 Study selection

Database searches identified 253 records, and nine additional reports were identified through other sources. After removing 56 duplicates, 197 records were screened and 169 were excluded. Of the 37 reports assessed for eligibility, 23 were excluded. Ultimately, 14 studies (13, 24–36) were included in the review (Figure 1).

**Figure 1:**
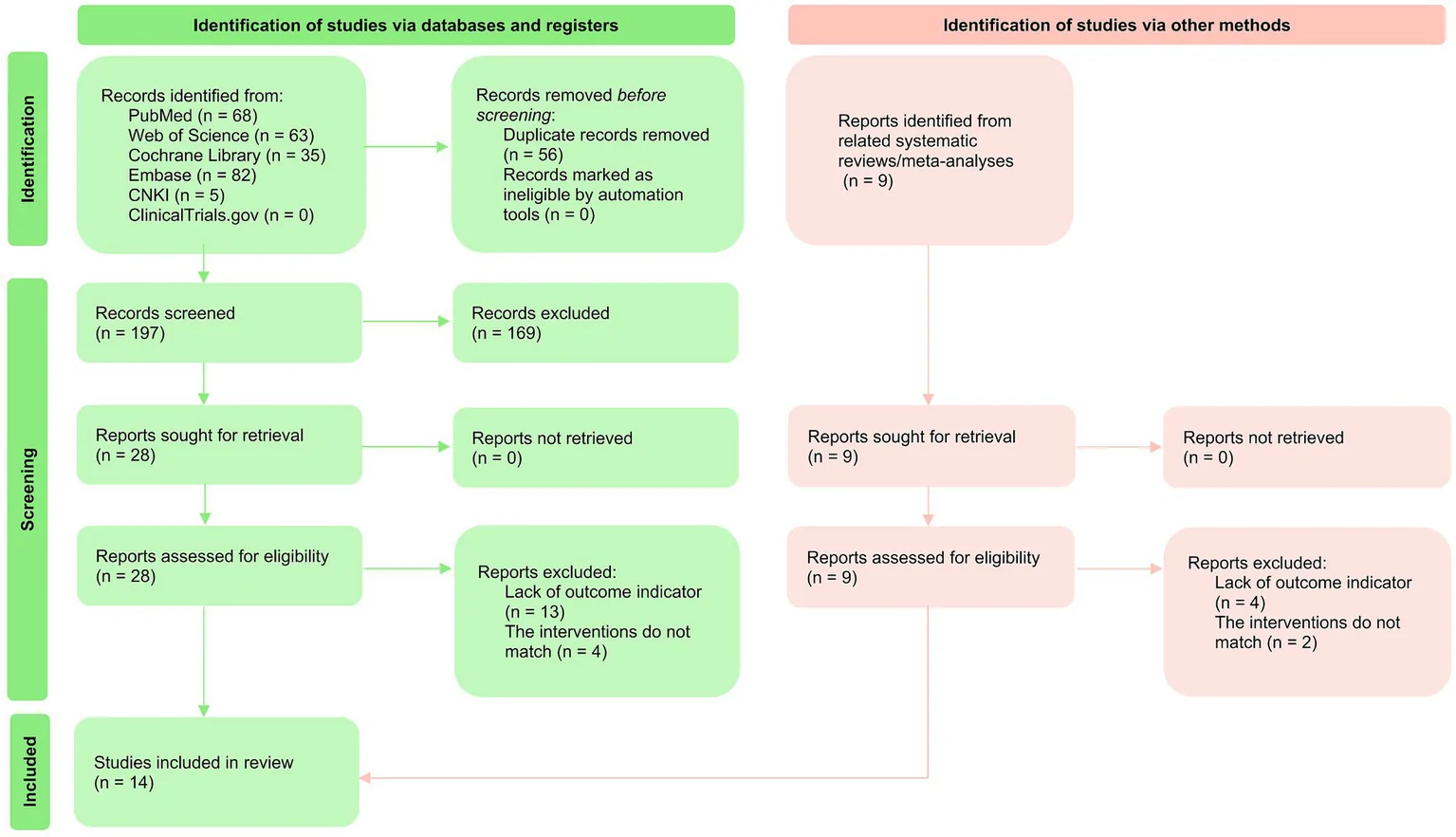
PRISMA 2020 flow diagram of study selection.

### 3.2 Risk of bias in studies

Most studies were judged at low risk in the domains of random sequence generation, incomplete outcome data, and selective reporting. For blinding of participants and personnel (performance bias), most studies were judged at high risk. For allocation concealment and blinding of outcome assessment (detection bias), some studies were rated as unclear risk due to insufficient reporting. A small number of studies were also judged at high risk in the “other bias” domain (Figure 2).

**Figure 2:**
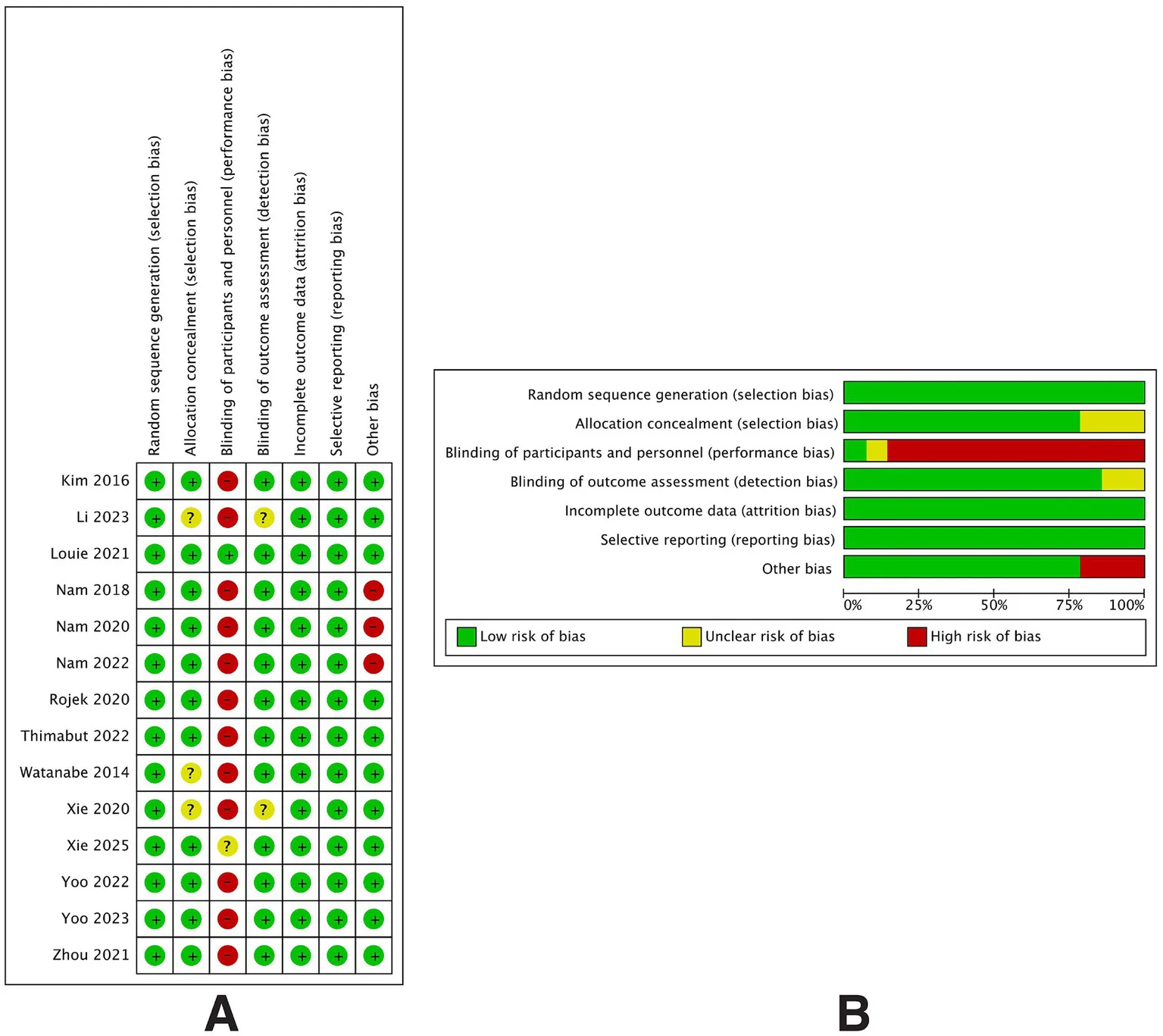
Risk of bias assessment of included randomized controlled trials (Cochrane RoB 1). **(A)** Risk of bias summary (traffic-light plot). **(B)** Risk of bias graph (percentage of studies per domain).

### 3.3 Study characteristics

The main characteristics of the included studies are summarised in Table 1. Fourteen RCTs (total sample size 565) published between 2014 and 2025 were included, predominantly from Asian countries (China, South Korea, Japan, and Thailand), with additional studies from Canada and Poland. Sample sizes were generally small, with most studies including approximately 8–30 participants per group. Mean participant age ranged from approximately 47 to 70 years. Time since stroke ranged from the subacute to chronic phase, although reporting formats varied across studies.

**Table 1:** Characteristics of included randomized controlled trials of wearable lower-limb exoskeleton-assisted gait training in stroke survivors.

Author year (Ref.)	Country	Sample size (T/C)	Age (T/C)	Disease duration / Stroke phase (T/C)	Type of intervention (T/C)	Duration of intervention	Main outcome	Robot type
Watanabe et al. (2014) (24)	Japan	11/11	67 ± 16.8/75.6 ± 13.9	58.9 ± 46.5/50.6 ± 33.8 days (acute/subacute; phase not explicitly reported)	the single-leg version of the Hybrid Assistive Limb/Conventional gait training	4 weeks twelve 20-min sessions	FAC, TUG, 6MWT, LE Fugl-Meyer	HAL
Kim et al. (2019) (25)	Korea	9/8	48.7 ± 7.01/46 ± 15.64	21.2 ± 33.27/10.22 ± 8.54 months (subacute; phase not explicitly reported)	robot-assisted gait training combined withconventional physical therapy/conventional physical therapy	4 weeks (comprising 20 sessions in total, 5 sessions per week)	BBS, LE Fugl-Meyer, FAC, 10MWT	Lokomat robotic-orthosis
Nam (2019) (26)	Korea	18/16	48.33 ± 15.56/68.56 ± 17.35	580.11 ± 389.21/284.81 ± 309.04 days (mixed subacute/chronic)	Electromechanical-assisted gait training using the exoskeleton device, alongside traditional physical therapy/Physical therapist-assisted gait training by conventional method, alongside traditional physical therapy	4 weeks 30 min each day, 5 days a week	FAC, RMI, 10MWT, 6MWT, MI, MBI, BBS	Exowalk. It is an electromechanical exoskeleton device designed for overground walking exercise without the need for a harness for weight support.
Nam et al. (2020) (27)	Korea, China	18/20	60 ± 11.48/57.3 ± 8.71	545.67 ± 295.94/600.45 ± 505.62 days (chronic)	Electromechanically assisted gait training/Physical therapist-assisted gait training	60 min/day, 5 days/week, for 2 weeks	FAC, 10MWT, 6MWT, MI, BBS	Exowalk
Rojek et al. (2020) (28)	Poland	23/21	69 ± 8/70 ± 6	120–365/150–365 days (subacute to chronic)	Ekso GT exoskeleton-assisted gait training + occupational therapy + physical therapy/Conventional rehabilitation	4 weeks (5 sessions per week, 45 min of exoskeleton training per session)	RMI, MBI	Ekso GT
Xie et al. (2020) (36)	China	30/30	65.53 ± 14.02/56.23 ± 17.86	87.62 ± 50.34/73.51 ± 42.84 days (subacute)	Conventional rehabilitation training + Lower limb exoskeleton robot training/Conventional rehabilitation training only	8 weeks, 5 times per week, 20 min per session	LE Fugl-Meyer, BBS, FAC, 6MWT, 10MWD, TUG	Lower limb exoskeleton rehabilitation robot (Model: Aiwalker, Beijing Ai Robot Technology Co., Ltd.)
Louie et al. (2021) (29)	Canada	19/17	59.6 ± 15.8/55.3 ± 10.6	36.7 ± 19.0/40.9 ± 19.8 months (chronic)	Exoskeleton-based physical therapy (EksoGT), replacing 75% of standard physical therapy sessions, focusing on standing and stepping repetition/ Standard physical therapy (without exoskeleton)	Until discharge or up to a maximum of 8 weeks	BBS, FAC, 6MWT, LE Fugl-Meyer	EksoGT (Ekso Bionics, USA)
Zhou et al. (2021) (34)	China	30/30	47.25 ± 4.92/48.64 ± 5.15	52.5 ± 16.2/47.4 ± 18.6 days (subacute)	Conventional physical therapy + mobile lower limb exoskeleton robot training / Conventional physical therapy (including gait training)	4 weeks, 6 sessions per week	BBS, 10MWT, LE Fugl-Meyer	Mobile lower limb exoskeleton robot (Model: AL800)
Nam et al. (2022) (30)	Korea	54/55	60.63 ± 15.61/62.42 ± 15.04	767.17 ± 1435.78/522.40 ± 1220.70 days (chronic; high variability)	Electromechanical-assisted gait training (EXOWALK) / Physical therapist-assisted gait training	30 min/day, 5 days/week for 4 weeks	FAC, RMI, 10MWT, 6MWT, MI, BBS, RMI	EXOWALK
Thimabut et al. (2022) (31)	Thailand	13/13	52.8 ± 12.6/62.8 ± 8.5	56.15 ± 23.71/72.54 ± 20.12 days (subacute)	Robot-assisted gait training (Welwalk) + physiotherapy + ground ambulation training/Physiotherapy + ground ambulation training only	30 sessions (5 days/week for 6 weeks)	6MWT	Welwalk (WW-1000), an exoskeleton-type robot-assisted gait training device
Yoo (2023) (32)	Korea	9/8	61 (42–85) / 65 (43–87)	19 (10–30) / 43 (11–119) months (chronic)	Overground RAGT using exoskeleton + conventional therapy / Conventional gait training + conventional therapy	30 min/session, 3 times/week, for 4 weeks	BBS, FAC, 10MWT, TUG, MBI, LE Fugl-Meyer	ExoAtlet Medy (powered exoskeleton for hip and knee joints)
Yoo (2023) (33)	Korea	15/15	61.86 ± 7.10/66.00 ± 5.31	11.57 ± 7.49/15.80 ± 7.14 months (chronic)	Healbot G (wearable powered exoskeleton) assisted treadmill training/Treadmill training alone	30 min per session, 10 sessions per week, for 4 weeks	BBS, FAC, 10MWT, MI	Healbot G (wearable powered exoskeleton, developed by T-ROBOTICS, actuating hip and knee joints)
Li et al. (2023) (35)	China	20/20	51.80 ± 11.06/45.75 ± 11.27	51.20 ± 17.42/46.45 ± 19.75 days (subacute)	Exoskeleton robot-assisted rehabilitation training / Conventional lower limb rehabilitation training	40 min/day, 6 days/week, for 3 weeks	BBS, FAC, MBI, LE Fugl-Meyer	ProWalk Exoskeleton Robot (Shenzhen CHVAS Technology Co., Ltd.)
Xie et al. (2025) (13)	China	30/30	61.63 ± 5.90/61.80 ± 5.55	8 (5,16) / 7 (5,13) weeks (early post-stroke; subacute)	Bilateral soft exoskeleton-assisted treadmill training / Conventional treadmill training	20 days (30 min of treadmill training + 30 min of conventional physical therapy daily)	BBS, FAC, LE Fugl-Meyer	Yrobot Relink™ bilateral soft exoskeleton system (Yrobot Inc., China)

T, Trial group; C, Control group; 10MWT, 10-Meter Walk Test; 6MWT, 6-Minute Walk Test; BBS, Berg Balance Scale; FAC, Functional Ambulation Category; TUG, Timed Up and Go test; RMI, Rivermead Mobility Index; MI-LE, Motricity Index–Lower Extremity; MBI, Modified Barthel Index; FMA-LE, Fugl–Meyer Assessment of the Lower Extremity.

The included interventions demonstrated substantial clinical and technical heterogeneity. Wearable lower-limb robotic exoskeleton-assisted gait training was delivered using different robotic systems, including rigid and soft exoskeletons, unilateral and bilateral assistance configurations, and different training environments (overground versus treadmill/platform-based training). The requirement for additional external support (eg, body-weight support systems, walkers, crutches, or therapist assistance), robotic assistance strategies, and training protocols also varied across studies. Detailed intervention characteristics are summarised in Supplementary Table S1.

Interventions mainly involved lower-limb exoskeleton- or robot-assisted gait training combined with conventional rehabilitation, whereas control groups generally received usual rehabilitation/usual gait training or dose-matched control training. However, comparator interventions also varied in rehabilitation content, intensity, and therapist involvement when reported, reflecting differences in the underlying rehabilitation context.

Primary outcomes were assessed across multiple clinically relevant domains, including walking independence (FAC), walking performance/endurance (10MWT; 6MWT), balance and functional mobility (BBS; TUG; RMI), lower-limb motor function (FMA-LE; MI-LE), and ADL (MBI/K-MBI).

### 3.4 Results of syntheses

#### 3.4.1 Effects on walking and balance outcomes

##### 3.4.1.1 FAC

Nine studies (13, 24–27, 30, 32, 33, 35) involving 359 participants (intervention group *n* = 185; control group *n* = 174) reported FAC, where higher scores indicate better walking independence. No statistical heterogeneity was observed across studies (τ^2^ = 0.0052; I^2^ = 0.0%; Q = 8.00, df = 8, *p* = 0.4337). Using the prespecified random-effects model, the pooled between-group estimate favoured the intervention group for FAC (MD = 0.39, 95% CI 0.15–0.63; *p* = 0.0053) (Figure 3A). The 95% prediction interval ranged from 0.10 to 0.69.

**Figure 3:**
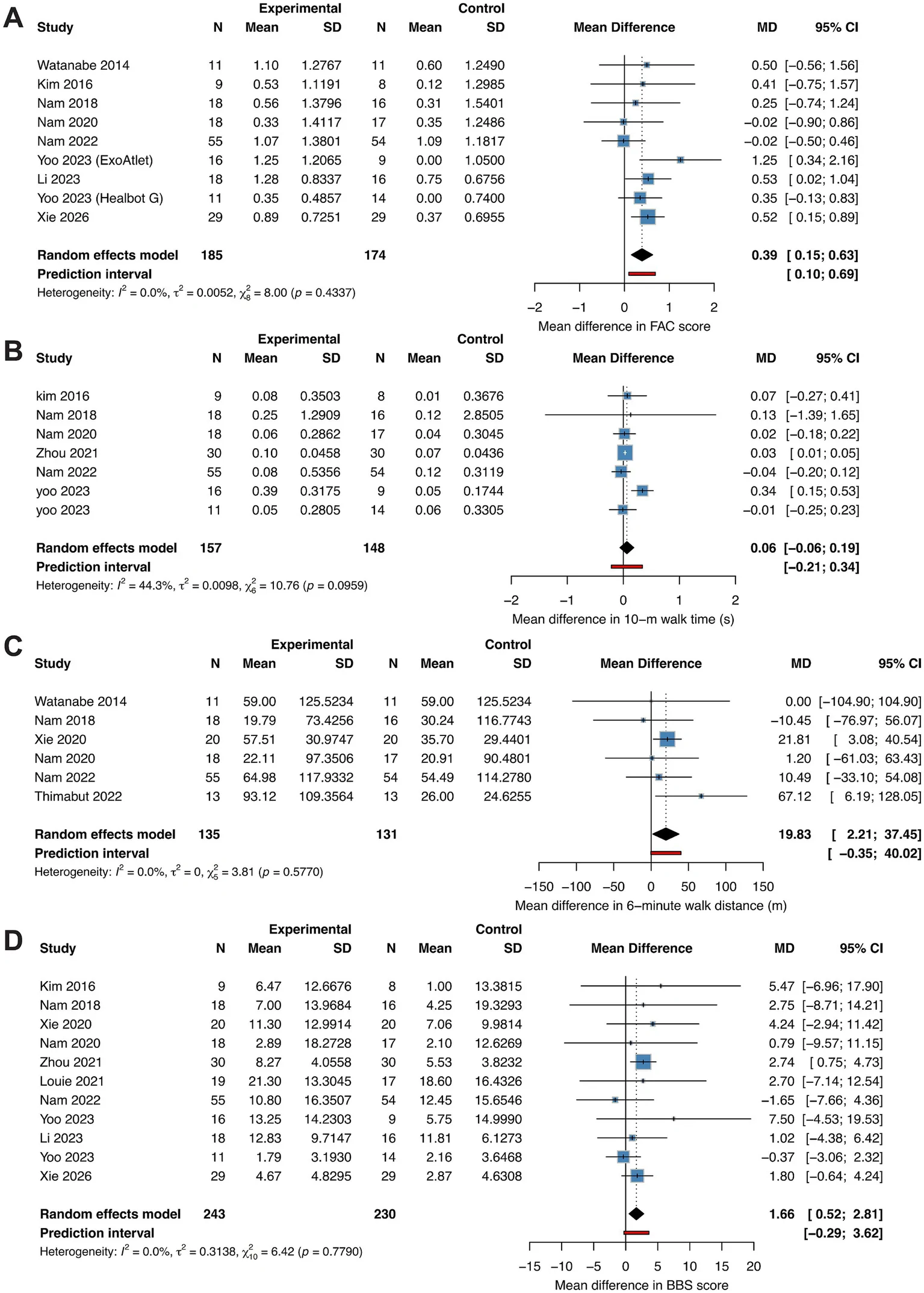
Forest plots of walking ability and performance outcomes (random-effects meta-analysis; REML with Hartung–Knapp 95% CI). **(A)** Functional Ambulation Category (FAC). **(B)** 10-Meter Walk Test (10MWT; time, s). **(C)** 6-Minute Walk Test (6MWT; distance, m). **(D)** Berg Balance Scale (BBS).

##### 3.4.1.2 10MWT

Seven studies (25–27, 30, 32–34) involving 305 participants (intervention group *n* = 157; control group *n* = 148) reported the 10MWT (10-m walking time, where shorter times indicate better walking performance). There was moderate heterogeneity across studies (τ^2^ = 0.0098; I^2^ = 44.3%; Q = 10.76, df = 6, *p* = 0.0959). Using the prespecified random-effects model, no statistically significant difference was observed in 10MWT time between the exoskeleton/robot-assisted gait training group and the control group (MD = 0.06 s, 95% CI − 0.06 to 0.19; *p* = 0.2586) (Figure 3B). The 95% prediction interval ranged from −0.21 to 0.34.

##### 3.4.1.3 6MWT

Six studies (24, 26, 27, 30, 31, 36) involving 266 participants (intervention group *n* = 135; control group *n* = 131) reported the 6MWT (walking distance, where longer distances indicate better walking endurance/function). No statistical heterogeneity was observed across studies (τ^2^ = 0; I^2^ = 0.0%; Q = 3.81, df = 5, *p* = 0.5770). Using the prespecified random-effects model, the intervention group demonstrated a significantly greater 6MWT distance compared with the control group (MD = 19.83 m, 95% CI 2.21–37.45; *p* = 0.0341) (Figure 3C). The 95% prediction interval ranged from −0.35 to 40.02.

##### 3.4.1.4 BBS

Eleven studies (13, 25–27, 29, 30, 32–36) involving 473 participants (intervention group *n* = 243; control group *n* = 230) reported the BBS, where higher scores indicate better balance. No statistical heterogeneity was observed across studies (τ^2^ = 0.3138; I^2^ = 0.0%; Q = 6.42, df = 10, *p* = 0.7790). Using the prespecified random-effects model, the intervention group demonstrated significantly higher BBS scores compared with the control group (MD = 1.66 points, 95% CI 0.52–2.81; *p* = 0.0089) (Figure 3D). The 95% prediction interval ranged from −0.29 to 3.62.

#### 3.4.2 Effects on functional mobility, motor function, and ADL outcomes

##### 3.4.2.1 TUG

Three studies (24, 32, 36) involving 87 participants (intervention group *n* = 47; control group *n* = 40) reported the TUG (where shorter completion times indicate better functional mobility performance). Substantial heterogeneity was observed across studies (τ^2^ = 625.8025; I^2^ = 81.6%; Q = 10.87, df = 2, *p* = 0.0044). Using the prespecified random-effects model, no statistically significant difference was observed in TUG time between the intervention and control groups (MD = −13.22 s, 95% CI − 82.53 to 56.08; *p* = 0.4979) (Figure 4A). The 95% prediction interval ranged from −139.60 to 113.15.

**Figure 4:**
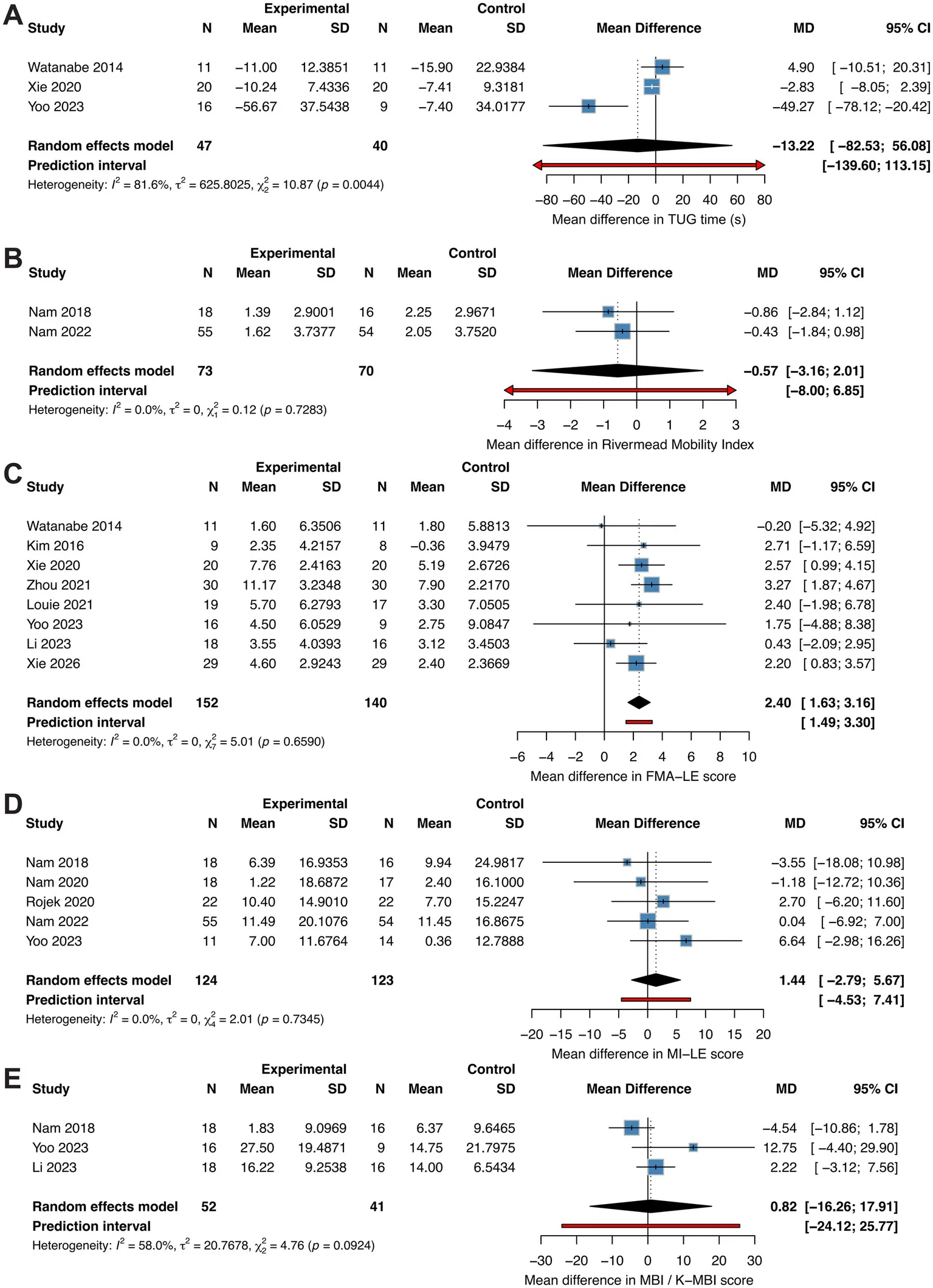
Forest plots of balance, functional mobility, motor function, and ADL outcomes (random-effects meta-analysis; REML with Hartung–Knapp 95% CI). **(A)** Timed Up and Go (TUG; s). **(B)** Rivermead Mobility Index (RMI). **(C)** Fugl–Meyer Assessment—Lower Extremity (FMA-LE). **(D)** Motricity Index—Lower Extremity (MI-LE). **(E)** Modified Barthel Index (MBI/K-MBI).

##### 3.4.2.2 RMI

Two studies (26, 30) involving 143 participants (intervention group *n* = 73; control group *n* = 70) reported the RMI, where higher scores indicate better mobility. No statistical heterogeneity was observed across studies (τ^2^ = 0; I^2^ = 0.0%; Q = 0.12, df = 1, *p* = 0.7283). Using the prespecified random-effects model, no statistically significant difference was observed in RMI scores between the intervention and control groups (MD = −0.57, 95% CI − 3.16 to 2.01; *p* = 0.2163) (Figure 4B).

##### 3.4.2.3 FMA-LE

Eight studies (13, 24, 25, 29, 32, 34–36) involving 292 participants (intervention group *n* = 152; control group *n* = 140) reported the FMA-LE, where higher scores indicate better lower-limb motor function. No statistical heterogeneity was observed across studies (τ^2^ = 0; I^2^ = 0.0%; Q = 5.01, df = 7, *p* = 0.6590). Using the prespecified random-effects model, FMA-LE scores were higher in the intervention group than in the control group (MD = 2.40, 95% CI 1.63–3.16; *p* = 0.0001) (Figure 4C). The 95% prediction interval ranged from 1.49 to 3.30.

##### 3.4.2.4 MI-LE

Five studies (26–28, 30, 33) involving 247 participants (intervention group *n* = 124; control group *n* = 123) reported the MI-LE, where higher scores indicate better lower-limb strength/motor function. No statistical heterogeneity was observed across studies (τ^2^ = 0; I^2^ = 0.0%; Q = 2.01, df = 4, *p* = 0.7345). Using the prespecified random-effects model, no statistically significant difference was observed between the intervention and control groups in MI-LE scores (MD = 1.44, 95% CI − 2.79 to 5.67; *p* = 0.3984) (Figure 4D). The 95% prediction interval ranged from −4.53 to 7.41.

##### 3.4.2.5 Modified Barthel Index/Korean Modified Barthel Index (MBI/K-MBI)

Three studies (26, 32, 35) involving 93 participants (intervention group *n* = 52; control group *n* = 41) reported MBI/K-MBI, where higher scores indicate better ADL. Moderate heterogeneity was observed across studies (τ^2^ = 20.7678; I^2^ = 58.0%; Q = 4.76, df = 2, *p* = 0.0924). Using the prespecified random-effects model, no statistically significant difference was observed between the intervention and control groups in total MBI/K-MBI scores (MD = 0.82, 95% CI − 16.26 to 17.91; *p* = 0.8547) (Figure 4E). The 95% prediction interval ranged from −24.12 to 25.77.

### 3.5 Sensitivity analyses

Leave-one-out sensitivity analyses were performed for TUG and MBI/K-MBI because of substantial heterogeneity or potential influence from individual studies. Sequential removal of individual studies did not materially alter the pooled estimates, and the overall conclusions remained unchanged (Tables 2, 3).

**Table 2:** Leave-one-out sensitivity analysis for TUG (MD, random-effects REML + Hartung–Knapp).

Omitted study	Pooled MD (s)	95% CI (s)	*p* value	τ^2^	I^2^ (%)
Watanabe et al. (2014) (24)	−23.79	−317.44 to 269.85	0.491	966.45	89.6
Xie et al. (2020) (36)	−20.76	−364.42 to 322.91	0.583	1327.97	90.5
Yoo et al. (2023) (32)	−2.03	−31.90 to 27.84	0.546	0.00	0.0

Leave-one-out sensitivity analysis for the Timed Up and Go test (TUG), using mean differences (MDs) and a random-effects model with restricted maximum likelihood (REML) estimation and Hartung–Knapp adjustment.

**Table 3:** Leave-one-out sensitivity analysis for MBI (MD, random-effects REML + Hartung–Knapp).

Omitted study	Pooled MD	95% CI	*p* value	τ^2^	I^2^ (%)
Nam (2019) (26)	4.20	−48.12 to 56.53	0.493	13.46	24.3
Yoo (2023) (32)	−0.94	−43.80 to 41.92	0.827	13.93	60.9
Li et al. (2023) (35)	2.19	−104.93 to 109.32	0.838	106.00	70.9

Leave-one-out sensitivity analysis for the Modified Barthel Index (MBI), using mean differences (MDs) and a random-effects model with restricted maximum likelihood (REML) estimation and Hartung–Knapp adjustment.

### 3.6 Subgroup analyses

To explore potential sources of heterogeneity, subgroup analyses were conducted according to training environment (overground versus treadmill/platform-based training) for outcomes with sufficient available studies. No statistically significant subgroup differences were observed for either FAC or BBS, suggesting that the training environment did not significantly modify the effects of exoskeleton-assisted gait training on walking ability or balance outcomes (Supplementary Figure S1).

### 3.7 Small-study effects and publication bias

For the outcome with at least 10 included studies (BBS in this review, *k* = 11), a contour-enhanced funnel plot was generated and Egger’s regression test was performed to evaluate potential small-study effects and publication bias. The contour-enhanced funnel plot showed no apparent asymmetry (Figure 5). Egger’s regression test did not indicate statistically significant small-study effects (*p* = 0.528) (37) Because all other outcomes included fewer than 10 studies, and given the limited reliability of funnel plots and regression tests in small-sample settings, publication bias assessments were not performed for the remaining outcomes.

**Figure 5:**
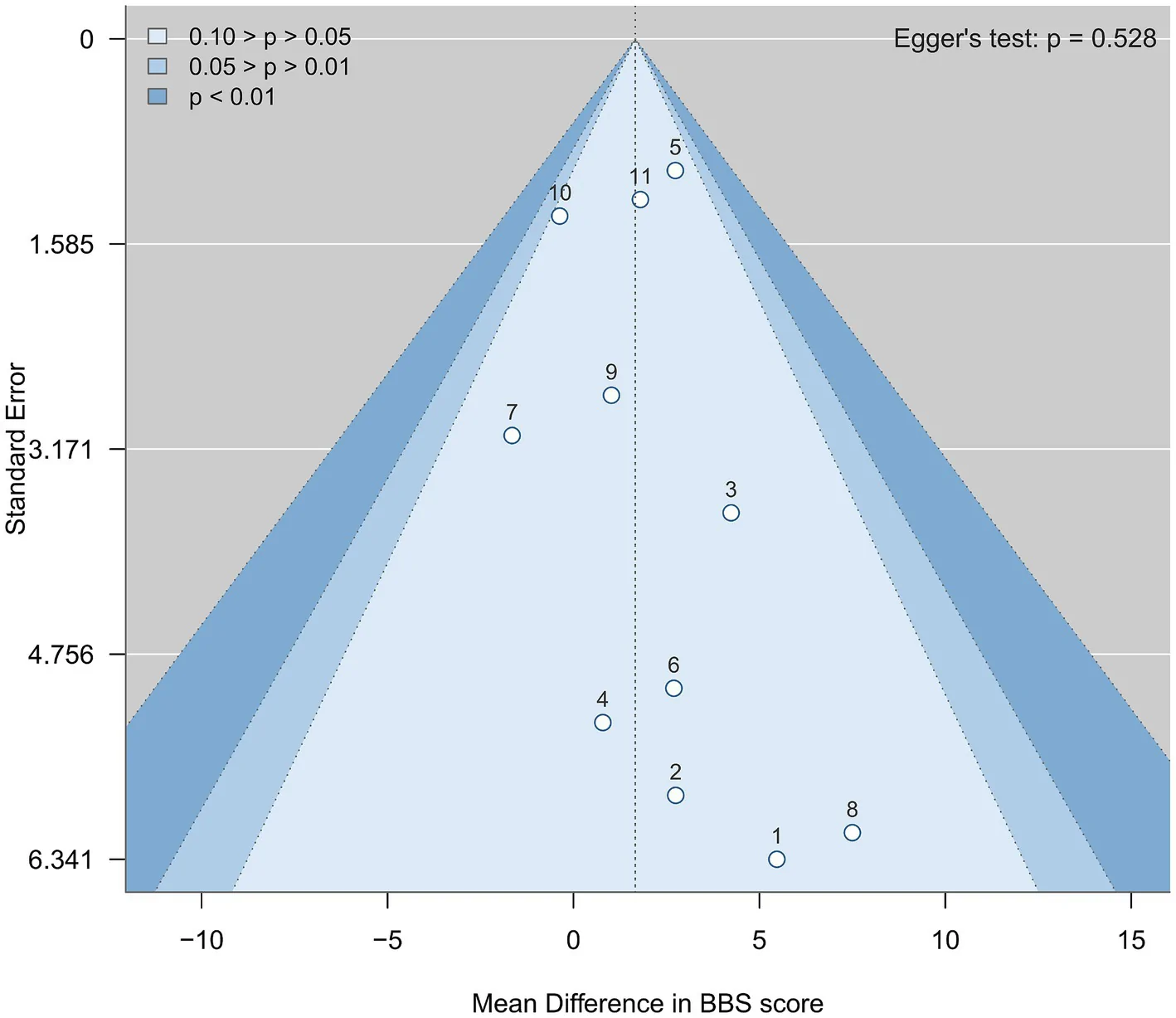
Contour-enhanced funnel plot for BBS (*k* = 11) with Egger’s regression test.

### 3.8 Certainty of evidence

The GRADE assessment indicated that the overall certainty of evidence ranged from moderate to very low (Table 4). Evidence certainty was moderate for walking independence (FAC), balance (BBS), and lower-limb motor function (FMA-LE); low for walking speed, walking endurance, and lower-limb strength outcomes (10MWT, 6MWT, and MI-LE); and very low for functional mobility and ADL outcomes (TUG, RMI, and MBI/K-MBI). Downgrading was mainly driven by risk of bias based on RoB 1, with additional downgrading for imprecision (few studies and wide CIs) and, for some outcomes, inconsistency (e.g., elevated I^2^). No evidence of small-study effects was detected for BBS (*k* = 11).

**Table 4:** GRADE evidence profile for the effects of exoskeleton-assisted training on walking ability, balance, and functional outcomes in stroke survivors (random-effects meta-analyses).

Outcome	Study design	Studies (participants)	Effect (random, MD [95% CI])	Risk of bias (RoB1)	Inconsistency	Indirectness	Imprecision	Publication bias	Overall certainty (GRADE)
FAC	RCT	9 (359)	0.39 [0.15, 0.63]	Serious	Not serious (I^2^ = 0%)	Not serious	Not serious	Not assessed (k < 10)	Moderate
10MWT	RCT	7 (305)	0.06 [−0.06, 0.19]	Serious	Not serious–Serious (I^2^ = 44.3%)	Not serious	Serious	Not assessed (k < 10)	Low
6MWT	RCT	6 (266)	19.83 [2.21, 37.45]	Serious	Not serious (I^2^ = 0%)	Not serious	Serious	Not assessed (k < 10)	Low
BBS	RCT	11 (473)	1.66 [0.52, 2.81]	Serious	Not serious (I^2^ = 0%)	Not serious	Not serious	Undetected (funnel + Egger, *p* > 0.05)	Moderate
TUG	RCT	3 (87)	−13.22 [−82.53, 56.08]	Serious	Very serious (I^2^ = 81.6%, τ^2^ very large)	Not serious	Very serious (very wide CI; crosses no effect)	Not assessed (k < 10)	Very low
RMI	RCT	2 (143)	−0.57 [−3.16, 2.01]	Serious	Not serious (I^2^ = 0%)	Not serious	Very serious (k = 2; CI crosses no effect)	Not assessed (k < 10)	Very low
FMA-LE	RCT	8 (292)	2.40 [1.63, 3.16]	Serious	Not serious (I^2^ = 0%)	Not serious	Not serious	Not assessed (k < 10)	Moderate
MI-LE	RCT	5 (247)	1.44 [−2.79, 5.67]	Serious	Not serious (I^2^ = 0%)	Not serious	Serious (CI crosses no effect)	Not assessed (k < 10)	Low
MBI/K-MBI	RCT	3 (93)	0.82 [−16.26, 17.91]	Serious	Serious (I^2^ = 58.0%)	Not serious	Serious (CI crosses no effect; wide CI)	Not assessed (k < 10)	Very low

Grading of Recommendations Assessment, Development and Evaluation (GRADE) evidence profile for the effects of exoskeleton-assisted training on walking ability, balance, and functional outcomes in stroke survivors (random-effects meta-analyses).

## 4 Discussion

This systematic synthesis of RCT evidence found that pooled between-group estimates favoured the intervention groups for walking independence (FAC), balance (BBS), and lower-limb motor control (FMA-LE), with moderate certainty of evidence for these outcomes. These estimates represent averages across clinically and technically heterogeneous intervention–comparator packages encompassing diverse robotic systems, training environments, assistance strategies, levels of patient engagement, training doses, and comparator content. In contrast, no clear pooled differences were observed for walking speed (10MWT), functional mobility (TUG), or ADL, for which the certainty of evidence was lower. Prediction intervals crossed the null for most outcomes, indicating that true effects may vary across clinical settings and patient populations. Consequently, the findings cannot determine whether the observed between-group differences are attributable to the robotic device category itself, identify which intervention components or patient groups account for these differences, or support device-specific or detailed prescribing recommendations.

Overall, consistent with previous systematic reviews and meta-analyses on post-stroke exoskeleton/robot-assisted gait training, we observed that the pooled estimates favoured the intervention groups for outcomes reflecting walking independence and functional grading. A Cochrane systematic review reported that electromechanical/robot-assisted gait training can increase the proportion of people with stroke who achieve independent walking, an outcome frequently measured using functional ambulation grading scales such as the FAC, indicating that functional ambulation scales may capture changes in walking independence and assistance requirements that are not immediately reflected in speed-based walking metrics (4). Meta-analyses of robot-assisted gait training have also reported overall effect directions indicating improvements in FAC and balance outcomes such as the BBS (38). More recently, systematic reviews and meta-analyses focusing on wearable robotic exoskeletons suggest that exoskeleton-assisted training may improve multiple walking-related outcomes, including walking ability, performance, and endurance, with potential benefits in balance- and motor function-related outcomes observed in some studies (7). However, these findings should be interpreted cautiously because wearable exoskeleton-assisted interventions encompass diverse device architectures, training environments, assistance strategies, and rehabilitation contexts. Differences between overground and treadmill/platform-based systems may influence the type of walking practice provided and should not be considered interchangeable. Therefore, compared with simple speed- or time-based metrics, outcomes such as FAC that more directly reflect achieving independent walking or requiring less assistance may provide complementary information regarding walking independence and assistance requirements.

It should be noted that no clear pooled differences were observed for performance-based or complex task outcomes such as walking speed (10MWT) and composite functional mobility (TUG), which may reflect limited statistical power, differences in outcome sensitivity, intervention heterogeneity, or genuinely variable or absent effects across settings. This finding differs from some previous reviews that have reported improvements in certain walking-related outcomes. For example, previous systematic reviews of wearable exoskeleton-assisted training have reported potential improvements in walking-related outcomes, although substantial variability remains across outcomes, devices, and study designs (8). The variability between studies may be explained by several factors. First, wearable exoskeleton-assisted training represents a heterogeneous intervention category, including different device architectures, training environments (overground versus treadmill/platform-based), assistance strategies, training doses, levels of patient engagement, comparator content, and rehabilitation contexts. These differences may influence the type and intensity of walking practice provided and may contribute to variability in outcomes requiring higher levels of independent locomotor control, such as walking speed and complex mobility tasks (4, 8, 10, 38). Second, differences in post-stroke stage and baseline functional capacity may influence treatment responsiveness. Walking speed and TUG require not only repeated gait practice but also sufficient residual motor control, balance, turning ability, and strength reserve. Variability in stroke stage and baseline walking ability across trials may therefore contribute to heterogeneous responses and limit the detection of consistent short-term improvements (4, 8, 10). Third, the present review applied conservative statistical approaches, including random-effects models with Hartung–Knapp adjustment and prediction intervals, to account for uncertainty and between-study variability. Particularly when the number of available studies is limited, these approaches may result in wider confidence and prediction intervals, providing a more cautious interpretation of potential clinical effects (17, 19, 39). Similarly, the unclear effect for ADL (MBI/K-MBI) is consistent with the concept that improvements in motor impairment or walking-related outcomes may not immediately translate into gains in self-care performance over short intervention periods. Future studies should include longer follow-up and explicitly assess functional transfer (4, 10).

From a neurorehabilitation and biomechanics perspective, the observed pattern of effects—relatively consistent improvements in FMA-LE and BBS, with greater uncertainty for speed, TUG, and ADL—may be biologically plausible; however, this interpretation should consider the substantial heterogeneity of exoskeleton-assisted training paradigms, including differences in device architecture, training environment, assistance strategies, and patient engagement. Wearable lower-limb robotic exoskeleton-assisted training, when appropriately configured, may provide opportunities for repetitive and task-specific gait practice, which could enhance sensorimotor input and support motor recovery processes. This interpretation aligns with theoretical frameworks in which post-stroke motor recovery depends on neuroplasticity and sensorimotor adaptation. Furthermore, abnormal synergies and associated movements are closely related to walking function and are reflected in impairment measures such as the Fugl–Meyer Assessment. Therefore, improvements in selective lower-limb control and coordinated gait performance may be more readily detected by impairment-level outcomes such as FMA-LE. For balance, post-stroke asymmetrical weight bearing and impaired postural control can restrict dynamic balance control. These impairments may reduce effective centre-of-mass transfer and limit paretic-limb support during walking. Some exoskeleton-assisted training configurations may increase opportunities for practicing paretic-side loading, weight-shift control, and gait-related postural adjustments, potentially contributing to improvements in balance-related measures such as BBS. Consistently, previous systematic reviews/meta-analyses often report that exoskeleton/robot-assisted gait training improves balance and walking independence-related outcomes. By contrast, speed/complex mobility tasks/ADL depend more heavily on self-propulsion, power generation, and integration of multiple motor and environmental demands. Walking speed is closely related to forward propulsion, whereas differences in device mechanics, assistance strategies, gait progression, and active participation requirements may contribute to variability in speed-related outcomes across studies. Moreover, TUG and ADL outcomes require more complex functional abilities, including turning, sit-to-stand transitions, upper-limb use, and adaptation to daily environments. According to the the International Classification of Functioning, Disability and Health framework, these outcomes reflect real-world performance rather than only functional capacity measured under structured training conditions. Therefore, improvements in impairment-level or balance outcomes may not immediately translate into better daily-life functioning. Additional task-specific training and longer follow-up may be required to facilitate functional transfer.

This review provides outcome-oriented information on wearable lower-limb robotic exoskeleton-assisted gait training after stroke, but does not support prescriptive guidance for its clinical use. Overall, pooled estimates favoured the intervention groups for walking independence (FAC), balance (BBS), and lower-limb motor control (FMA-LE), whereas estimates for walking speed (10MWT) and ADL (MBI/K-MBI) remained uncertain. However, these estimates represent average contrasts across heterogeneous intervention–comparator packages rather than effects attributable to the robotic device category itself. The influences of device architecture, training environment, body-weight support, assistance strategy, patient engagement, training dose, comparator content, and patient selection could not be resolved. Finally, outcome assessment should balance sensitivity and clinical relevance: FAC provides information about walking independence and the level of physical assistance required (40), whereas BBS reflects postural control; speed and ADL outcomes reflect different functional dimensions and may also vary with training dose, follow-up duration, and individual variability. Given that prediction intervals crossed the line of no effect for multiple outcomes, the current evidence cannot identify which patients, devices, or training conditions account for the observed between-group differences and does not support device-specific, patient-specific, or detailed prescribing recommendations. Future trials should use standardised intervention reporting and adequately powered stratified analyses to identify the intervention components and patient characteristics associated with outcomes.

Although this review followed a preregistered protocol and standard procedures for systematic reviews and meta-analyses, several limitations remain, largely reflecting the scale, reporting completeness, and clinical and technical heterogeneity of the primary studies. First, although this review focused on wearable lower-limb robotic exoskeleton-assisted gait training, the included interventions represented heterogeneous technological and therapeutic paradigms rather than a single uniform treatment modality. Trials differed substantially in device architecture, training environment (overground versus treadmill/platform-based training), assistance strategies, control modes, requirements for external support (eg, body-weight support systems, walkers, crutches, or therapist assistance), patient engagement, and training dose parameters (frequency, session duration, and total intervention period). These differences may partly contribute to variability in treatment effects, particularly for complex functional outcomes such as TUG. However, more granular subgroup analyses according to device architecture, assistance mode, body-weight support, external walking aids, patient engagement, or dose parameters were not feasible because the number of available RCTs was limited and reporting of these intervention characteristics was inconsistent across studies. Second, participants spanned a wide range of post-stroke stages and baseline functional levels (from subacute to chronic), and recovery potential and responsiveness to rehabilitation may differ across stages and baseline ambulatory abilities. Therefore, pooling heterogeneous populations may reduce the ability to identify potentially differential effects in specific subgroups, such as individuals in the subacute stage or those with different levels of baseline walking ability. Third, most studies assessed outcomes immediately post-intervention, with limited follow-up at 3–6 months or longer, restricting our ability to determine whether observed benefits are sustained over time or whether functional decline occurs after discontinuation of training. Fourth, for some outcomes (particularly ADL and TUG), the number of studies and sample sizes were limited. Even with random-effects models incorporating Hartung–Knapp adjustment and prediction intervals, uncertainty intervals may remain wide. Prediction intervals crossing the line of no effect suggest that true effects may vary across clinical contexts, and external generalisation should therefore be interpreted cautiously. Finally, reporting of measurement implementation details for some outcomes (eg, 10MWT/TUG test protocols, whether assistive devices were permitted, and assessor blinding) was insufficient. Publication bias and small-study effects could not be systematically assessed beyond outcomes with ≥10 studies. Furthermore, comparator interventions were variably described, and differences in usual rehabilitation content, therapist involvement, and training intensity may have influenced the observed between-group contrasts. Therefore, the pooled estimates should be interpreted as average between-group contrasts across heterogeneous exoskeleton-assisted intervention packages and heterogeneous comparator conditions, rather than as effects attributable to robotic assistance itself or as a direct comparison between otherwise equivalent robotic and non-robotic gait training. Together, these factors reduce the certainty and clinical interpretability of the available evidence.

## 5 Conclusion

This systematic review and meta-analysis of available RCT evidence found that, across wearable lower-limb robotic exoskeleton-assisted interventions encompassing both overground and treadmill/platform-based implementations, pooled between-group estimates favoured the intervention groups for walking independence (FAC), balance (BBS), and lower-limb motor control/impairment (FMA-LE). However, the effects on walking speed (10MWT), functional mobility (TUG), and ADL (MBI/K-MBI) remain uncertain, indicating that favourable estimates at the impairment and walking-independence levels may not consistently translate into broader functional performance. The pooled estimates should be interpreted cautiously because prediction intervals crossed the line of no effect for most outcomes, suggesting variability across patient populations and intervention characteristics. These estimates represent average contrasts across heterogeneous intervention–comparator packages, and the influences of device architecture, training environment, body-weight support, assistance strategy, patient engagement, training dose, comparator content, and patient selection could not be resolved. Therefore, the observed between-group differences cannot be attributed to the robotic device category itself, and the current evidence does not support device-specific, patient-specific, or detailed prescribing recommendations. Future studies should use larger, well-designed RCTs with standardised intervention reporting, stratified analyses, and longer follow-up to identify which intervention components and patient characteristics are associated with outcomes.

## Data Availability

The original contributions presented in the study are included in the article/Supplementary material, further inquiries can be directed to the corresponding author.
