# Supplementary material for: Effects of wearable lower-limb robotic exoskeleton–assisted gait training on balance, walking, and activities of daily living after stroke: a systematic review and meta-analysis
Source: Front Neurol. 2026 Oct 16;17:1818981. doi: 10.3389/fneur.2026.1818981 (PMC13719558; doi:10.3389/fneur.2026.1818981)
Supplement: Supplementary file 1 [file Supplementary_file_1.docx]

Appendix A

| Line | Concept | PubMed |
| --- | --- | --- |
| 1 | Stroke population terms | ("Stroke"[Mesh] OR stroke*[tiab] OR cerebrovascular accident*[tiab] OR "cerebral infarction"[tiab] OR "intracerebral hemorrhage"[tiab] OR poststroke[tiab] OR post-stroke[tiab]) |
| 2 | Exoskeleton / wearable robot | (exoskeleton*[tiab] OR "robotic exoskeleton*"[tiab] OR "powered exoskeleton*"[tiab] OR "lower limb exoskeleton*"[tiab] OR exosuit*[tiab] OR "soft exosuit*"[tiab] OR "wearable robot*"[tiab] OR "wearable robotic*"[tiab] OR "robot-assisted gait"[tiab] OR "robotic gait"[tiab] OR "robot-assisted walking"[tiab] OR "robotic-assisted walking"[tiab] OR "Hybrid Assistive Limb"[tiab] OR HAL[tiab] OR "Honda Walking Assist"[tiab] OR "Honda walking assist device"[tiab] OR Ekso[tiab] OR "Ekso Bionics"[tiab] OR ReWalk[tiab] OR Indego[tiab] OR "ExoAtlet"[tiab] OR MINDWALKER[tiab] OR "robotics"[Mesh] OR "Orthotic Devices"[Mesh]) |
| 3 | Randomized trials | (randomized controlled trial[pt] OR controlled clinical trial[pt] OR random*[tiab] OR randomly[tiab] OR trial[tiab] OR placebo[tiab] OR "clinical trial"[tiab] OR "clinical trials as topic"[Mesh]) |
| 4 | Exclude animals | NOT (animals[mh] NOT humans[mh]) |
| 5 | Final | ( #1 AND #2 AND #3 ) #4 |

| Line | Concept | Web of Science |
| --- | --- | --- |
| 1 | Stroke population terms | TS=(stroke* OR "stroke" OR "cerebrovascular accident" OR "cerebral infarction" OR "intracerebral hemorrhage" OR poststroke OR post-stroke) |
| 2 | Exoskeleton / wearable robot | TS=(exoskeleton* OR exosuit* OR "soft exosuit*" OR ("robotic" NEAR/2 exoskeleton*) OR ("powered" NEAR/2 exoskeleton*) OR ("lower limb" NEAR/2 exoskeleton*) OR ("wearable" NEAR/3 robot*) OR ("wearable" NEAR/3 robotic*) OR "hybrid assistive limb" OR HAL OR "Honda Walking Assist" OR "Honda walking assist device" OR Ekso OR "Ekso Bionics" OR ReWalk OR Indego OR ExoAtlet OR MINDWALKER) |
| 3 | Randomized trials (RCT/CCT signal) | TS=(randomi* OR random* OR randomly OR placebo* OR "controlled trial*" OR "clinical trial*" OR trial OR trials OR group* OR "double blind" OR "single blind" OR crossover OR cross-over) |
| 4 | Final set | #1 AND #2 AND #3 |

| Line | Concept | Cochrane Library |
| --- | --- | --- |
| 1 | Stroke population terms | MeSH descriptor: [Stroke] explode all trees |
| 2 | Stroke (text words) | (stroke OR "cerebrovascular accident" OR "cerebrovascular apoplexy" OR "brain infarction" OR "cerebral infarction" OR "intracerebral hemorrhage" OR "intracranial hemorrhage" OR poststroke OR post-stroke):ti,ab,kw |
| 3 | Stroke set | #1 OR #2 |
| 4 | Exoskeleton / wearable robot (text words) | (exoskeleton* OR "robotic exoskeleton*" OR "powered exoskeleton*" OR "lower limb exoskeleton*" OR exosuit* OR "soft exosuit*" OR "wearable robot*" OR "wearable robotic*" OR "robot-assisted gait" OR "robot-assisted walking" OR "robotic gait" OR "robotic-assisted walking" OR "Hybrid Assistive Limb" OR HAL OR "Honda Walking Assist" OR "Honda walking assist device" OR Ekso OR "Ekso Bionics" OR ReWalk OR Indego OR ExoAtlet OR MINDWALKER):ti,ab,kw |
| 5 | Exoskeleton/robotics (MeSH, optional broaden) | MeSH descriptor: [Robotics] explode all trees |
| 6 | Exoskeleton set | #4 OR #5 |
| 7 | Trial keywords | (random* OR trial* OR placebo* OR "controlled trial" OR "clinical trial"):ti,ab,kw |
| 8 | Final | #3 AND #6 |
| 9 | Final + trial keywords | #3 AND #6 AND #7 |

| Line | Concept | Embase |
| --- | --- | --- |
| 1 | Stroke population terms | 'stroke'/exp  OR  (stroke* OR 'cerebrovascular accident*' OR 'cerebral infarction' OR 'brain infarction' OR 'intracerebral hemorrhage' OR 'intracranial hemorrhage' OR poststroke OR post-stroke) |
| 2 | Exoskeleton / wearable robot | ('exoskeleton'/exp OR exoskeleton*:ti,ab,kw OR exosuit*:ti,ab,kw OR (soft NEXT/1 exosuit*):ti,ab,kw OR (robotic NEAR/2 exoskeleton*):ti,ab,kw OR (powered NEAR/2 exoskeleton*):ti,ab,kw OR (lower NEAR/1 limb NEAR/2 exoskeleton*):ti,ab,kw OR (wearable NEAR/3 robot*):ti,ab,kw OR (wearable NEAR/3 robotic*):ti,ab,kw OR (robot NEAR/1 assisted NEAR/1 gait):ti,ab,kw OR (robot NEAR/1 assisted NEAR/1 walking):ti,ab,kw OR (robotic NEAR/1 assisted NEAR/1 walking):ti,ab,kw OR (hybrid NEAR/1 assistive NEAR/1 limb):ti,ab,kw OR HAL:ti,ab,kw OR (honda NEAR/1 walking NEAR/1 assist*):ti,ab,kw OR ekso:ti,ab,kw OR rewalk:ti,ab,kw OR indego:ti,ab,kw OR exoatlet:ti,ab,kw OR mindwalker:ti,ab,kw) |
| 3 | RCT filter | (('randomized controlled trial'/de OR 'controlled clinical trial'/de OR 'randomization'/de OR 'double blind procedure'/de OR 'single blind procedure'/de OR 'crossover procedure'/de OR 'placebo'/de) OR (random* OR randomi* OR randomly OR placebo* OR trial* OR crossover):ti,ab,kw OR ((controlled NEXT/1 trial*) OR (clinical NEXT/1 trial*) OR (double NEXT/1 blind) OR (single NEXT/1 blind) OR (cross NEAR/1 over)):ti,ab,kw) |
| 4 | Combine sets | #1 AND #2 AND #3 |
| 5 | Exclude animal-only | NOT ('animal'/exp NOT 'human'/exp) |
| 6 | Final | (#1 AND #2 AND #3) NOT ('animal'/exp NOT 'human'/exp) |

| Line | Concept | Field | CNKI |
| --- | --- | --- | --- |
| 1 | Population (Stroke population terms) | 主题 | 脑卒中 + 卒中 + 中风 + 脑血管意外 + 脑梗死 + 脑缺血 + 缺血性卒中 + 脑出血 + 出血性卒中 + 后卒中 + 卒中后 |
| 2 | Intervention (Exoskeleton) | 主题 | 外骨骼 + 外骨骼机器人 + 外骨骼式 + 穿戴外骨骼 + 下肢外骨骼 + 外骨骼康复机器人 |
| 3 | Study design (RCT) | 主题 | 随机 + 随机对照 + 随机抽样 + 随机对照试验 + 随机对照研究 |
| 4 | Final | — | #1 AND #2 AND #3 |

Supplementary Table S1. Taxonomy of included wearable lower-limb robotic exoskeleton-assisted gait training interventions

| Study | Device/system | Device type | Training environment | Assistance/control strategy | External support/walking aids | Training dose | Comparator characteristics |
| --- | --- | --- | --- | --- | --- | --- | --- |
| Watanabe 2014 | Hybrid Assistive Limb (HAL) | Rigid powered wearable exoskeleton; unilateral | Overground | Cybernic voluntary control; active assistance | Mobile suspension system; harness/support when required | Individualized training; frequency/duration as reported | Conventional gait training |
| Kim 2016 | Lokomat® | Treadmill-based robotic orthosis | Treadmill/platform-based | Robotic guidance force; adjustable assistance | Harness with partial body-weight support | RAGT sessions combined with conventional physiotherapy | Conventional physiotherapy |
| Nam 2018 | EXOWALK® | Wearable robotic exoskeleton | Overground | Electromechanical assistance; bilateral assistance | Walker/cane support; no routine harness reported | Protocol reported in study | Conventional gait training |
| Nam 2020 | EXOWALK® | Wearable robotic exoskeleton | Overground | Electromechanical assistance; bilateral | NR | Protocol reported in study | Conventional rehabilitation/gait training |
| Louie 2021 | EksoGT® (Ekso Bionics) | Rigid powered bilateral lower-limb exoskeleton | Overground | Powered assistance; individualized parameters | Assistive support as required during training | Exoskeleton-based physical therapy program | Usual rehabilitation |
| Rojeck 2020 | EksoGT® | Powered wearable exoskeleton | Overground | Powered exoskeleton-assisted gait training | Walker/crutches limitations described; details limited | Study protocol reported | Conventional rehabilitation |
| Nam 2022 | EXOWALK® | Wearable robotic exoskeleton | Overground | Electromechanical assistance | NR | Protocol reported | Conventional gait training |
| Thimabut 2022 | Welwalk WW-1000 | Robotic gait training device | Treadmill/platform-based | Adjustable robotic assistance | Body-weight support and safety devices | 30 sessions reported | Physiotherapy control |
| Yoo 2022 | Powered exoskeleton | Wearable powered exoskeleton | Overground | Powered assistance; individualized training | Mobile suspension system; walker/cane when needed | Individualized walking practice | Conventional rehabilitation |
| Yoo 2023 | Powered exoskeleton | Wearable powered exoskeleton | Overground gait training | Powered exoskeleton assistance | NR | Training protocol reported | Conventional rehabilitation |
| Xie 2026 | Yrobot Relink™ | Soft wearable exoskeleton | Treadmill walking | Soft exoskeleton assistance | Body-weight support system | 30 min/day; 20 days | Conventional treadmill walking |
| Chinese study 2021 | Mobile lower-limb exoskeleton robot | Wearable lower-limb exoskeleton | NR | NR | NR | Rehabilitation training protocol reported | Conventional exercise therapy |
| Chinese study 2020 (Xie) | AiWalker® exoskeleton robot | Wearable lower-limb exoskeleton | Walking training | Coordinated hip/knee/ankle assistance | NR | Training protocol reported | Conventional rehabilitation |
| Chinese study 2023 (Li) | Unilateral wearable exoskeleton robot | Wearable unilateral exoskeleton | NR | Unilateral assistance | NR | Training protocol reported | Conventional rehabilitation |

NR, not reported.

**A. Functional Ambulation Category (FAC)**

**Supplementary Figure S1. Subgroup analyses by training environment.** (A) Functional Ambulation Category (FAC). Studies were grouped into overground or treadmill/platform-based training. Diamonds indicate pooled subgroup estimates, horizontal lines indicate 95% confidence intervals, and red bars indicate prediction intervals. Positive mean differences favour exoskeleton-assisted gait training. Test for subgroup differences: P = 0.5554.

MD, mean difference; CI, confidence interval; SD, standard deviation.

**B. Berg Balance Scale (BBS)**

**Supplementary Figure S1 (continued). Subgroup analyses by training environment.** (B) Berg Balance Scale (BBS). Studies were grouped into overground or treadmill/platform-based training. Diamonds indicate pooled subgroup estimates, horizontal lines indicate 95% confidence intervals, and red bars indicate prediction intervals. Positive mean differences favour exoskeleton-assisted gait training. Test for subgroup differences: P = 0.2123.

MD, mean difference; CI, confidence interval; SD, standard deviation.
